# Silver-decorated gel-shell nanobeads: physicochemical characterization and evaluation of antibacterial properties

**DOI:** 10.3762/bjnano.11.49

**Published:** 2020-04-14

**Authors:** Marta Bartel, Katarzyna Markowska, Marcin Strawski, Krystyna Wolska, Maciej Mazur

**Affiliations:** 1 University of Warsaw, Department of Chemistry, Pasteura 1, 02-093 Warsaw, Poland; 2 University of Warsaw, Department of Biology, Miecznikowa 1, 02-093 Warsaw, Poland

**Keywords:** *Escherichia coli*, gel-shell particles, minimum biofilm inhibitory concentration (MBIC), minimum inhibitory concentration (MIC), nanocomposites, *Pseudomonas aeruginosa*, silver nanoparticles, *Staphylococcus sp*

## Abstract

We report on the synthesis of composite nanobeads with antibacterial properties. The particles consist of polystyrene cores that are surrounded by sulfonic gel shells with embedded silver nanoparticles. The nanocomposite beads are prepared by sulfonation of polystyrene particles followed by accumulation of silver ions in the shell layer and subsequent reduction with sodium borohydride. The resulting material has been characterized by electron microscopy, vibrational and X-ray photoelectron spectroscopy and several other experimental techniques. It was shown that sodium borohydride reduces silver ions embedded in the gel layer producing silver nanoparticles but also transforms a fraction of sulfonic groups in the polymer to moieties with sulfur in a lower oxidation state, likely thiols. It is hypothesized that the generated thiol groups are anchoring the nanoparticles in the gel shell of the nanobeads stabilizing the whole structure. The silver-decorated nanobeads appear to be a promising material with considerable antimicrobial activity and were tested against *Escherichia coli, Pseudomonas aeruginosa, Staphylococcus aureus* and *Staphylococcus epidermidis*. The determined minimum inhibitory (MIC) and minimum biofilm inhibitory (MBIC) concentrations are comparable to those of non-incorporated silver nanoparticles.

## Introduction

Over the last several years, scientific advances in synthetic polymer materials resulted in a number of applications. With regard to this, special attention has been paid to polymer nanoparticles [1–2]. Polymer nanobeads have been used in various areas including electrochemistry [3–4], catalysis [5–7] and drug delivery [8–9]. The main advantages of such particles are large surface area and low density. The particles can be further modified to provide new properties to these materials. One modification is the incorporation of metal nanoparticles into the polymer beads [10–17]. The resulting composites exhibit a double function, they support the metal nanoparticles and prevent their aggregation. For example, polystyrene microspheres have been decorated with silver nanoparticles and were used as catalysts, Raman-enhancing materials, optoelectronic elements and biomedical devices [18–19].

Polystyrene beads are also a versatile material that can be quite easily functionalized with sulfonic groups. The particles are incubated with concentrated sulfuric acid at elevated temperature, which results in gradual etching of their surface. Through sulfonation, the –SO_3_H groups are substituted to the benzene rings of the polymer. In consequence, a gel layer of sulfonated polystyrene is formed around the intact polystyrene core. Such structures are called gel-shell particles (PSS) [20–21]. The gel layer is highly hydrophilic and contains negatively charged sulfonic groups. The gel shell can be utilized for the accumulation of cationic species, e.g., the accumulation of a monomer followed by further polymerization [2,22].

Polystyrene-based gel-shell particles have been also used for the accumulation of silver ions followed by their reduction to generate metallic nanoparticles embedded in the shell. Such structures have been demonstrated to reveal antibacterial and antifungal properties. For example, Zhao et al. prepared micrometer-sized hybrid particles in a multi-step preparation involving the sulfonation of polystyrene beads and the incorporation of [Ag(NH_3_)_2_]^+^ complexes, followed by the reduction of the silver precursor with polyvinylpyrrolidone [23]. The resulting composite was examined with regard to its antimicrobial properties against *Escherichia coli* and *Staphylococcus aureus* bacteria. Similarly, Liao et al. obtained polystyrene sulfonate beads modified with polyaniline followed by decoration with silver nanoparticles and demonstrated the considerable antibacterial activity of this material [24].

While the number of works on hybrid polymer/nanoparticle structures and their antibacterial activity is relatively limited, a great number of studies has been devoted to non-supported silver nanoparticles and their antimicrobial properties. For example, Martínez-Castañón and co-workers showed that spherical silver nanoparticles of 7 nm in size inhibit the growth of *E. coli* and *S. aureus* in concentrations of 6.25 and 7.5 μg/mL, respectively [25]. In another study, citrate-stabilized nanoparticles (average diameter of 9 nm) inhibited the growth of *E. coli* and *S. aureus* at 10 and 5 μg/mL, respectively [26].

We report herein on the synthesis of nanocomposites with antibacterial properties. The polystyrene nanobeads were modified with sulfonic groups followed by accumulation of silver ions in the generated hydrogel layer. Then, the silver ions were reduced using sodium borohydride, which resulted in the formation of silver nanoparticles. It has been demonstrated that during the reduction process also the sulfonic groups in the polymer have been partially reduced to thiol moieties. In consequence, the thiols interact with the silver nanoparticles anchoring them in the gel layer on the nanobeads. Moreover, it has been shown that the nanocomposite exhibits considerable antibacterial activity, comparable or superior to that of non-incorporated silver nanoparticles.

## Results and Discussion

### Preparation and physicochemical characterization of silver-decorated gel-shell nanobeads

**Figure 1:**
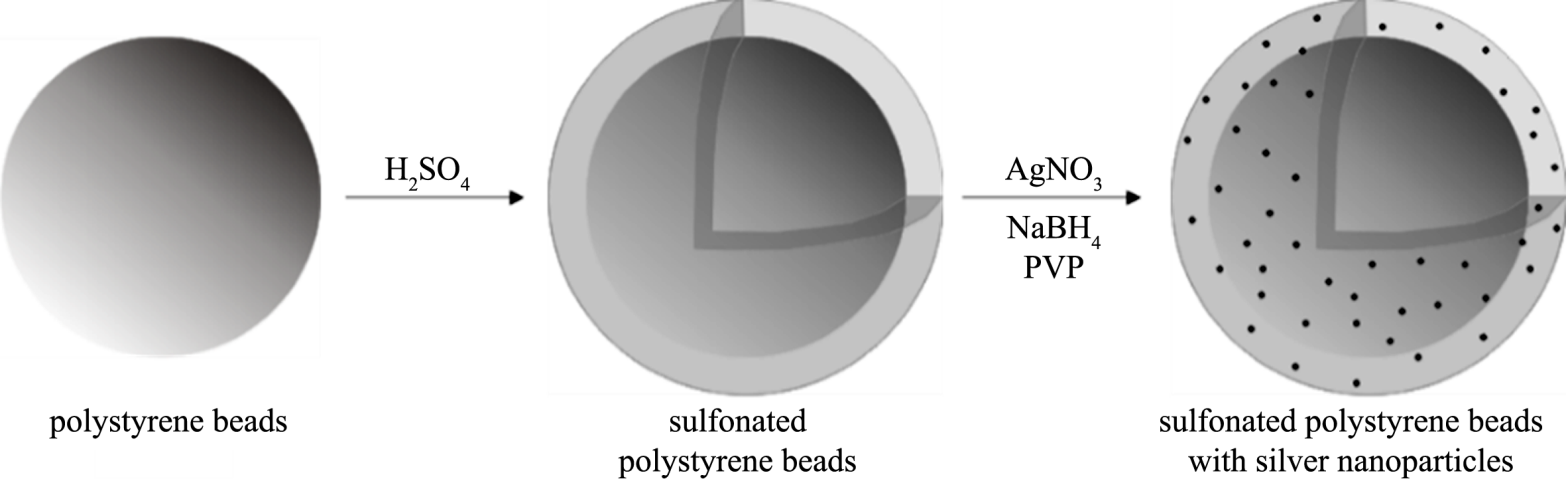
Scheme of the preparation of sulfonated polystyrene beads with embedded silver nanoparticles.

The preparation of sulfonated polystyrene beads with embedded silver nanoparticles (PSSAg) is schematically shown in Figure 1. First, polystyrene nanospheres are modified with sulfonic groups followed by accumulation of silver ions in the formed hydrogel layer. Then, the silver ions are reduced using sodium borohydride. The reaction proceeds concurrently within the gel layers of the particles and in the bulk of the solution. However, after completion of the reduction the non-incorporated nanoparticles are separated from the composite nanobeads through centrifugation. As a result, silver nanoparticles incorporated in the polymer beads are obtained.

**Figure 2:**
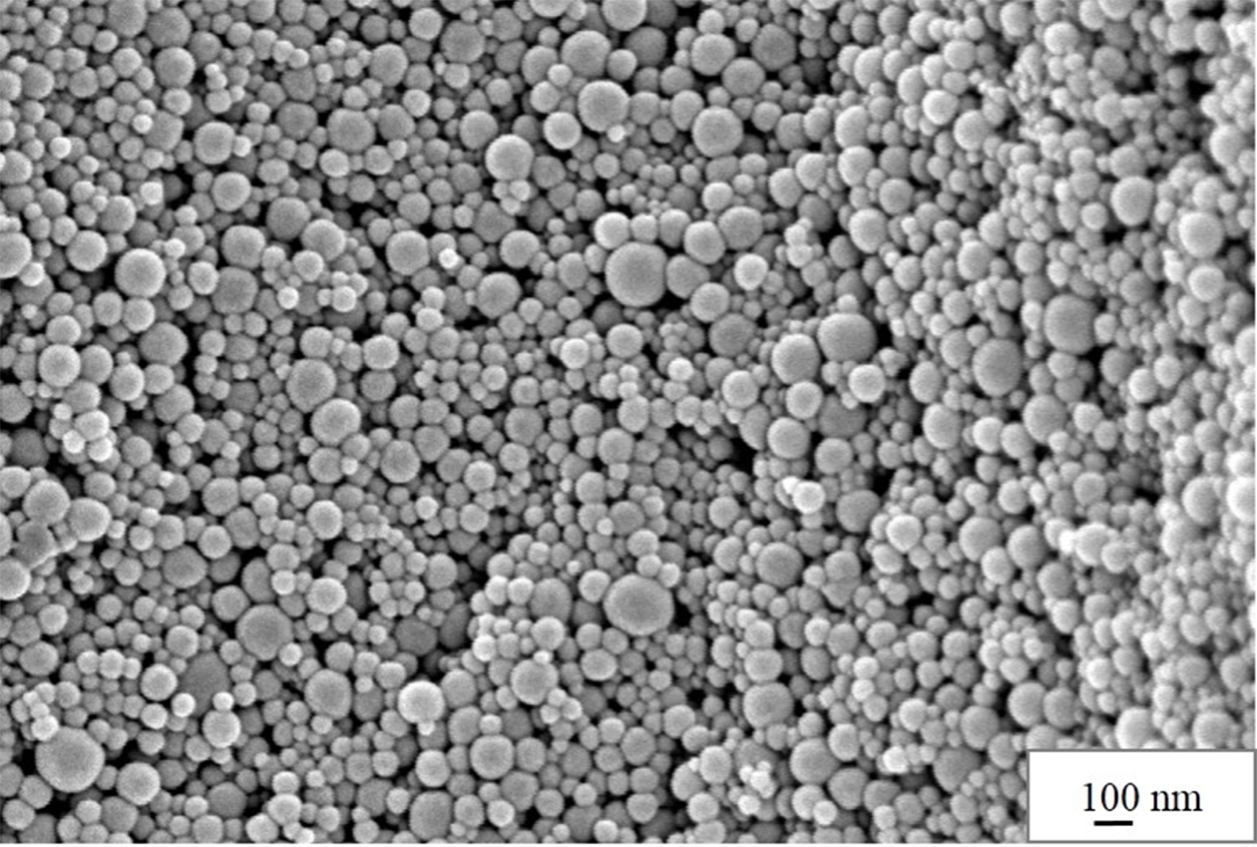
SEM image of sulfonated polystyrene beads.

In the first step, the polystyrene particles were reacted with concentrated sulfuric acid to incorporate sulfonate groups into the polymer. The resulting product was then examined using SEM. As seen in Figure 2, the beads exhibit a regular spherical shape with an average diameter of ca. 80 nm. A simple calculation assuming a nanobead density of 1 g/cm^3^ gives a specific surface area of ca. 75 m^2^/g. The morphology of the particles is similar to that of non-sulfonated polystyrene beads (SEM data not shown). Thus, if shape and size of the beads are retained during the reaction, the question arises whether they have been in fact transformed into the gel-shell particles [27–28].

**Figure 3:**
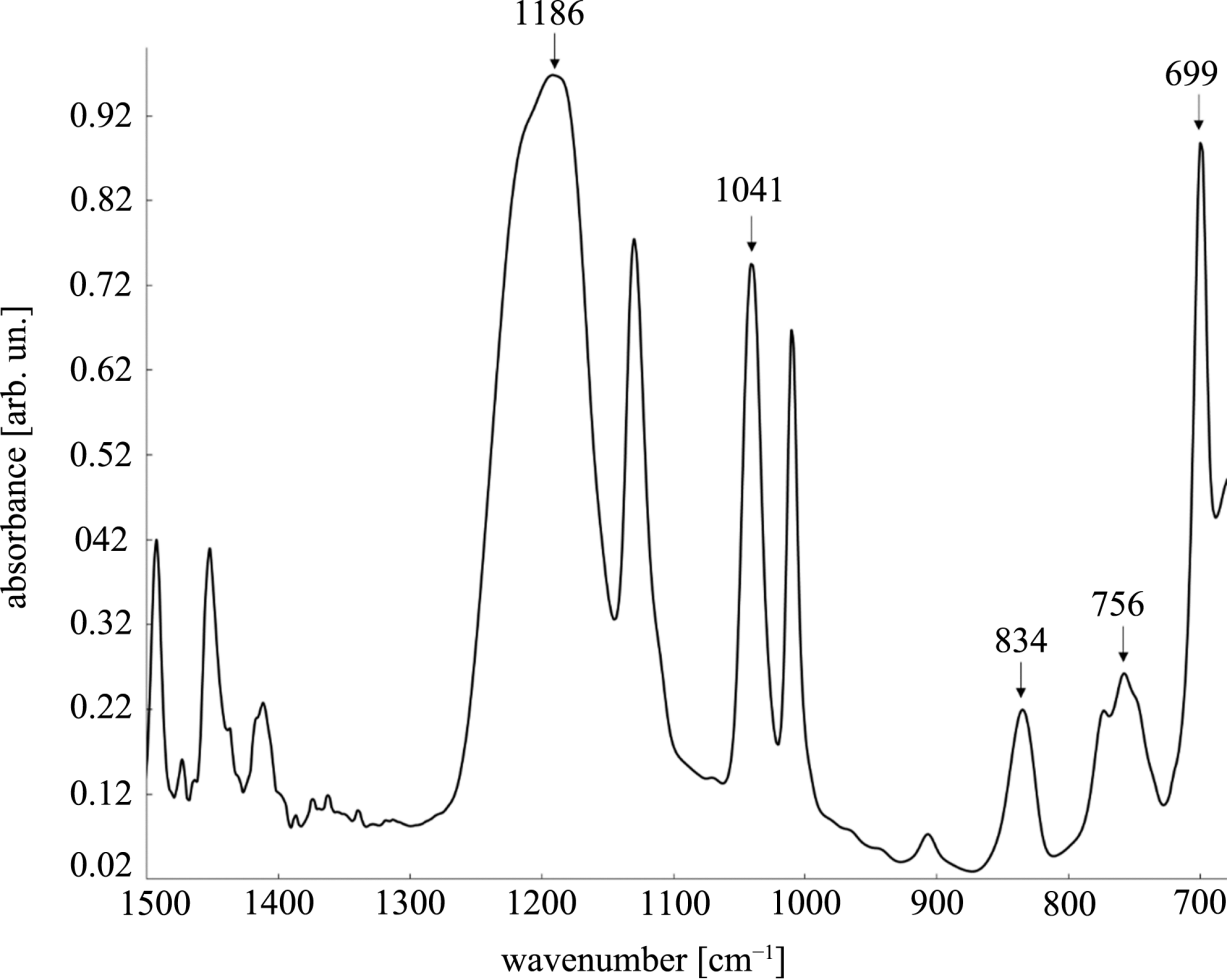
FTIR spectrum of sulfonated polystyrene nanobeads.

To answer this question FTIR spectroscopy has been employed. Figure 3 shows the FTIR spectrum of the sulfonated particles. The most intense mode at 1186 cm^−1^ with a broad shoulder is attributed to the asymmetric stretching of S=O bonds. The symmetric stretching vibrations of SO_3_^−^ groups produce the band at 1041 cm^−1^. The peak at 834 cm^−1^ is characteristic of C–H out-of-plane vibrations in *para*-substituted benzene rings. On the other hand, the 756 cm^−1^ mode is attributable to monosubstituted benzene due to the out-of-plane bending vibration of the five CH groups in the aromatic ring. The presence of these two bands confirms therefore that there is both sulfonated and non-sulfonated polystyrene. The peak at 699 cm^−1^ assigned to the out-of-plane skeleton bending vibrations of the benzene ring is correlated to the degree of sulfonation (the larger the intensity of the band, the lower the sulfonation degree). The FTIR results clearly confirm that the polystyrene beads are partially sulfonated [29].

In order to estimate the degree of sulfonation combustion elemental analysis has been carried out. The determined C/S ratio is 18:1, which corresponds to a degree of sulfonation of ca. 44%. A simple calculation (assuming a mean diameter of polystyrene beads of 80 nm) reveals that the thickness of the sulfonate gel layer is ca. 7 nm. This corresponds to ca. 22.3 sulfonic groups per square nanometer of the particle surface.

**Figure 4:**
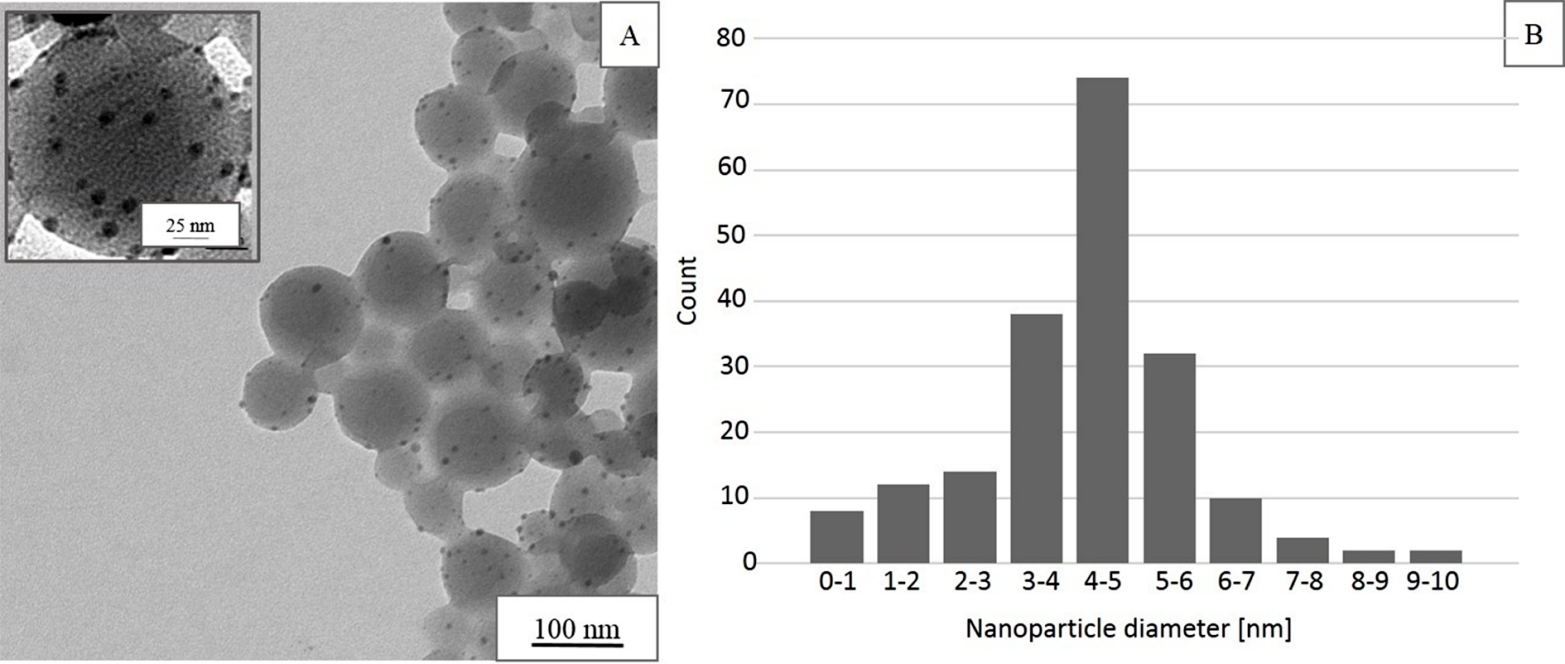
(A) TEM image of sulfonated polystyrene beads modified with silver nanoparticles and (B) size distribution histogram of silver nanoparticles estimated from the TEM image. Inset: magnification of an individual composite bead.

The next step in the preparative procedure was the incorporation of silver ions into the gel shell of the particles. The beads were incubated with silver ions with the intention that, through Coulomb interactions between anionic sulfonic groups and silver ions, the cations will accumulate in the hydrogel shell. Then, the reducing agent (sodium borohydride) was added to the reaction mixture to reduce the incorporated metal cations. After separation of the resulting material through centrifugation it was examined with TEM (Figure 4). The image clearly shows that the polymer particles retain their spherical shape and their size (ca. 80 nm) while being uniformly decorated with silver nanoparticles (seen as small black dots; the magnification of an individual nanosphere is shown as the inset in Figure 4). The average diameter of the nanoparticles is approximately 5 nm (a corresponding histogram of the size distribution is shown in Figure 4b).

Unfortunately, the TEM image does not allow us to discriminate whether the silver nanoparticles are distributed within the whole volume of the polymer beads or whether they are located exclusively in the gel shell. However, the latter case is much more likely since the polystyrene core of the beads is impermeable to water and ionic species. Therefore, it can be assumed that the silver nanoparticles are generated within the outer 7 nm gel shell of the particle while the polystyrene core is left intact.

**Figure 5:**
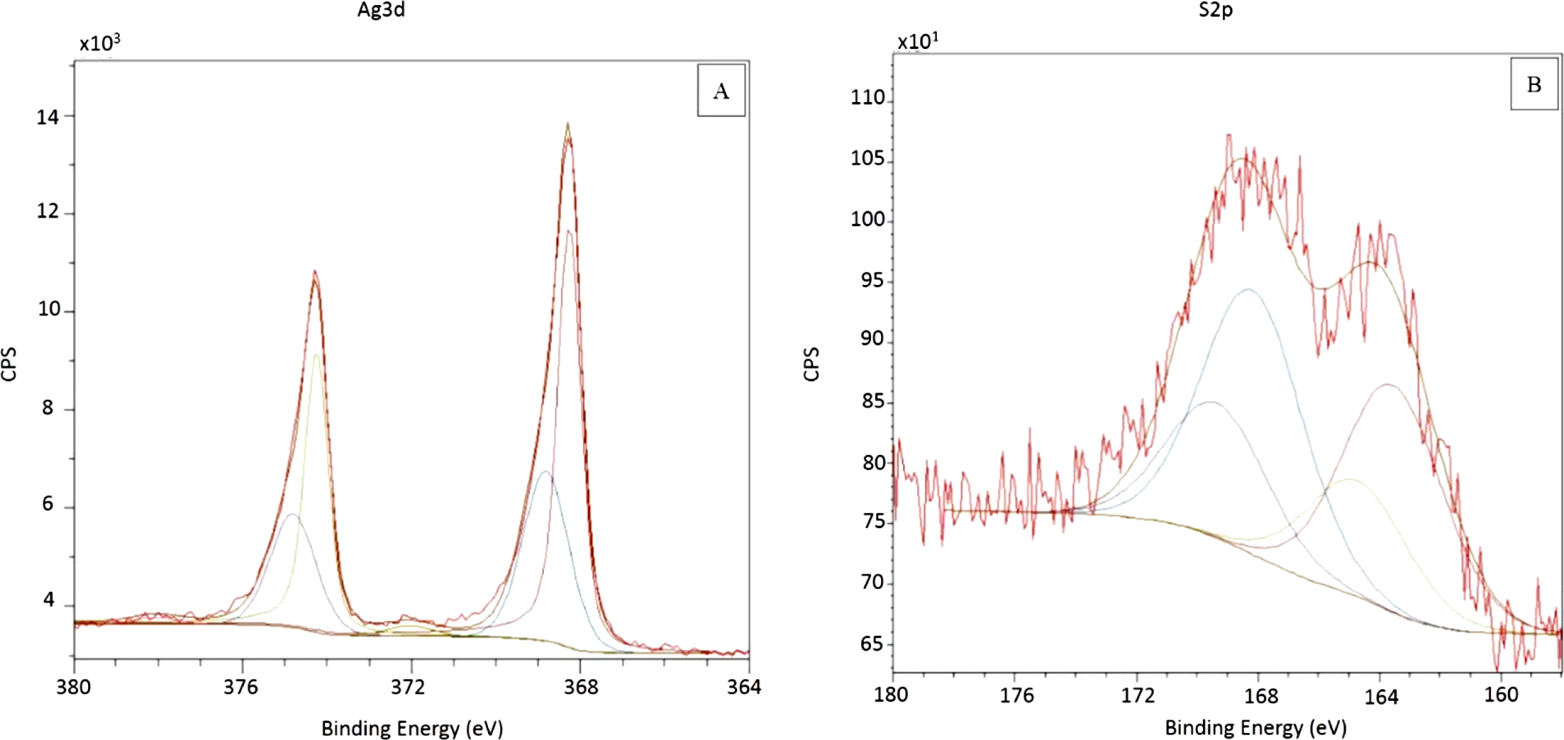
High-resolution XPS spectra of PSSAg nanobeads recorded over narrow ranges of binding energy: (A) Ag 3d, (B) S 2p region.

Next, the PSSAg nanobeads were studied with X-ray photoelectron spectroscopy (XPS). The spectrum confirms the presence of silver and sulfur in the sample. Figure 5 shows the high-resolution spectra for these elements. The direct evidence for metallic silver embedded in the polymer matrix is the spin–orbit doublet recorded at 368.3 and 374.3 eV for Ag 3d_5/2_ and 3d_3/2_, respectively (Figure 5A), followed by plasmon loss peaks at 372 and 378 eV [30–31]. However, the asymmetric shape of the spectra suggests another spin–orbit pair with binding energies at 368.8 and 374.8 eV. This indicates the presence of some other form of silver, e.g., Ag bonded to organic molecules [32] or non-reduced silver ions [33] embedded in the gel layer. The content of this form of silver is ca. 37.1% (w/w, with respect to the overall amount of Ag).

The S 2p signal reveals the presence of two non-equivalent types of sulfur atoms in the sample (the ratio of these two types of atoms is 1.2:1). The spin–orbit doublet (2p_3/2_/2p_1/2_) with a 2:1 intensity ratio and a binding energy splitting of 1.16 eV was used in modeling the data. The doublet at 169.4 and 168.2 eV can be assigned to the sulfonic form of sulfur [34–36]. This result is not surprising, since the reaction of polystyrene with sulfuric acid is intended to yield a substitution reaction in the benzene rings with sulfonic groups (this result has been confirmed by FTIR measurements). However, the contribution at lower binding energy (doublet at 164.7 and 163.5 eV) is unexpected. Such signals have not been reported for sulfonated polystyrene in the literature. The binding energy value suggests the presence of sulfur in a lower oxidation state. One should note that the reduction of silver ions is achieved by addition of sodium borohydride. It turns out that the sulfonic groups might have been reduced by this reducing agent to other sulfur species. Based on the binding energy value it can be suggested that the sulfonic group has been reduced to a thiol moiety [37]. If this is the case, it may be hypothesized that the generated thiols adsorb on the surface of the silver nanoparticles (the adsorption of thiols on silver surfaces is a well-known phenomenon). It should be noted, however, that the binding energy of sulfur in aromatic thiols adsorbed on a metal surface is ca. 162 eV [37]. As such a contribution is not detected in the XPS spectrum, it is possible that the number of thiol groups directly interacting with the surface of silver nanoparticles is low in comparison to the total amount of –SH moieties in the gel layer. On the other hand, the contribution at 368.8 eV observed in the Ag 3d signal (Figure 5a) may suggest that a fraction of silver atoms is interacting with thiol groups.

The analysis of the XPS data suggests an interesting scenario of the preparative process. Incubation of the gel-shell particles with silver ions results in the accumulation of the latter in the shell. The addition of the sodium borohydride results in a reduction of the metal ions but also in a reduction of the sulfonic groups. The metal ions are transformed to metallic silver in the form of nanoparticles, while a fraction of sulfonic groups is reduced to moieties of lower oxidation state, likely thiols. It seems that the thiol groups may interact with the surface of the nanoparticles anchoring them in the gel layer of the beads and stabilizing the whole structure. One should note that the interaction of silver with thiol groups is much stronger than that with sulfonic groups. Thus, the coordination of silver nanoparticles is preferred in terms of thiol rather than sulfonic groups.

**Figure 6:**
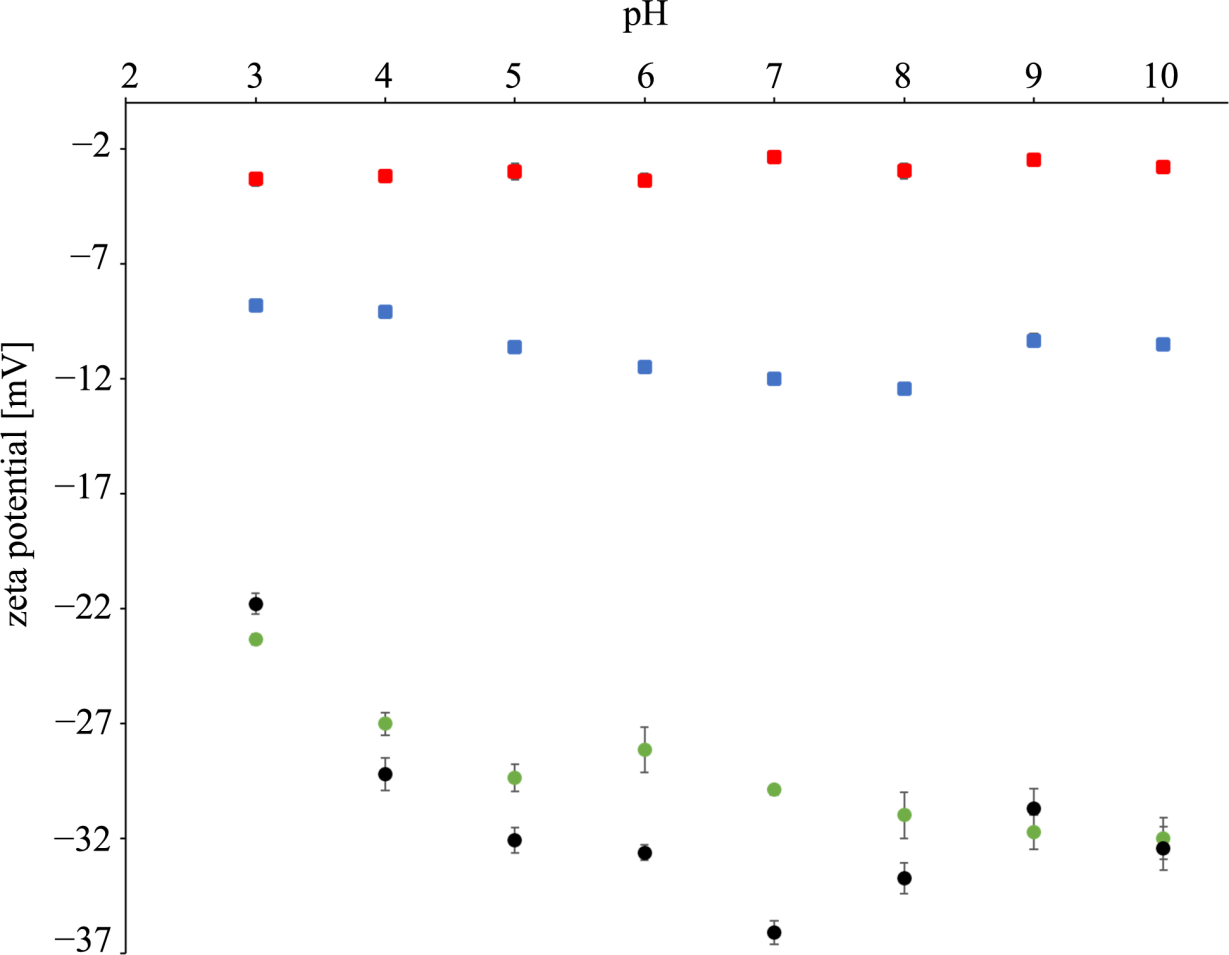
Zeta potential of PSS (green circles), PSS with silver ions (black circles), PSSAg (blue squares) and silver nanoparticles (red squares) as a function of the pH value.

If the above scenario is correct, one can deduce that, due to the conversion of ca. 55% of the sulfonic groups, the charge accumulated on the particles (due to anionic sulfonic groups) should be considerably diminished. To test this hypothesis, zeta potential measurements have been done. The analysis was performed in buffer solutions from pH 3 to pH 10 for the PSSAg beads, but also for PSS particles, PSS beads with incorporated silver ions and silver nanoparticles (non-incorporated in the polymer). The data is shown in Figure 6.

One can see that for the PSS and the PSS with incorporated Ag^+^ ions the obtained results are very similar. At high pH values, the zeta potential values are ca. −30 mV, while they gradually increase with increasing pH value. This can be explained assuming that in both cases the negative charge of sulfonic groups is neutralized by sodium or silver cations. With decreasing pH values the sulfonate groups are increasingly protonated and the absolute value of the zeta potential decreases. The zeta potential of PSSAg beads exhibits considerably lower absolute values (from −8.8 to −10.5 mV). This is understandable if one takes into account that during the reduction with sodium borohydride a fraction of sulfonic groups is being reduced to thiols. As thiol groups are not charged, the total negative charge on the PSSAg beads is decreased, which yields lower absolute values of the zeta potential [38]. One can also notice, that the zeta potential of the silver nanoparticles that are not embedded in polymer beads is close to zero. Thus, the presence of metallic Ag in the beads does not contribute significantly to the total value of the measured zeta potential. The important conclusion from the above discussion is that the zeta potential measurements indirectly confirm the XPS data on the reduction of sulfonic groups to non-ionic thiol species.

**Figure 7:**
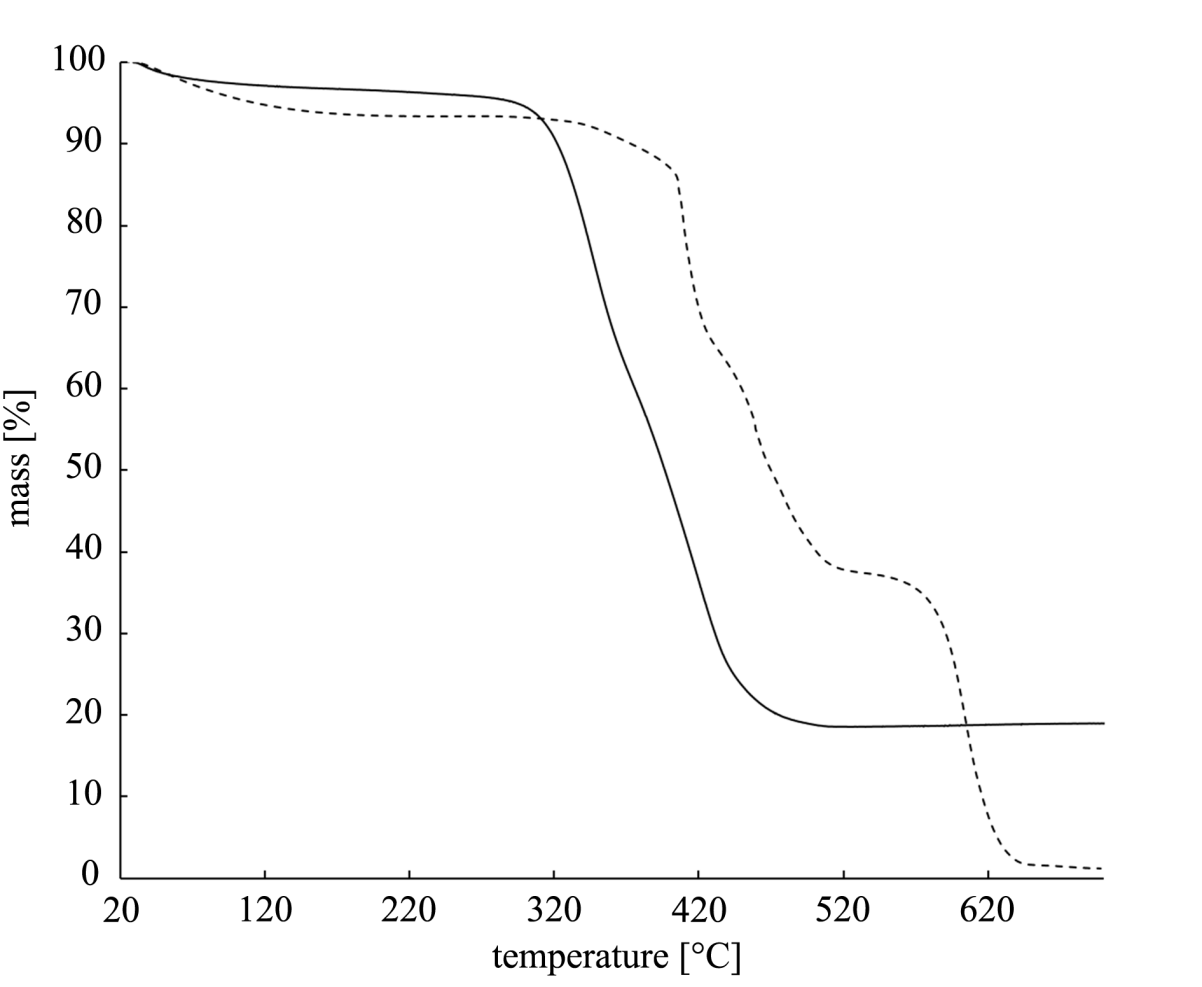
TGA thermograms of sulfonated PSS (dashed line) and PSSAg nanobeads (solid line).

The next step in the studies was the determination of the absolute content of silver in the nanocomposite using thermogravimetric analysis. The thermograms of PSS and PSSAg have been recorded up to 700 °C under oxygen/nitrogen atmosphere at a heating rate of 5 °C/min (Figure 7). For the PSS sample one can see several decomposition steps in the range of 400–650 °C, which is characteristic of sulfonated polystyrene [39–40]. The important information is that the mass decreases to a residual value of 1.2%. For the PSSAg sample the decomposition starts at a lower temperature (320–450 °C) and the final relative residual mass is ca. 18.6%. It seems that the difference in the TGA curves for PSS and PSSAg samples can be explained by a catalytic effect of silver. The difference between the relative residual masses of PSSAg and PSS is ca. 17.4%, which may be attributed to the silver content in the composite.

The proposed preparative protocol yields nanosized polymeric beads with well-dispersed silver nanoparticles. This approach is similar in some aspects to synthetic strategies reported in the literature. For example, Li et al. [41] proposed the decoration of the surface of polystyrene microspheres with silver nanoparticles through adsorption (the Ag nanoparticles were generated in the solution through reduction of AgNO_3_ with sodium borohydride). Since polystyrene is hydrophobic, the adsorption of hydrophilic Ag species in aqueous medium results in a decrease of the Gibbs surface free energy. The advantage of this method is its simplicity. However, even though it was demonstrated that the resulting structures are stable in aqueous solution, this may not be the case after addition of surfactants or the exchange of the solvent for a more hydrophobic one. Zhao et al. [23] proposed to incorporate silver nanoparticles into polystyrene/polystyrene sulfonate particles through accumulation of a silver complex in the gel shell followed by the reduction with polyvinylpyrrolidone. Since the PVP is a weak reducing agent, the reaction was carried out for prolonged times (several hours), which yielded micrometer-sized composite beads. In contrast to these examples, our approach allows one to fabricate stable composite particles (the Ag nanoparticles are coordinated to thiol groups generated in situ during the synthesis), in a simple and relatively fast reaction.

### Antibacterial activity of PSSAg nanocomposite

We were further interested whether the silver-modified beads show antibacterial effects. To determine the antibacterial potential of the synthesized material, precise broth micro-dilution assays have been performed for selected gram-negative and gram-positive bacteria, mostly pathogens. The obtained results are presented in Table 1. The concentrations are expressed as the mass of metallic silver contained in the composite per volume of the colloidal solution (to calculate the values, a silver content of 17.4% was assumed in the composite, as determined by thermogravimetric analysis).

**Table 1:** MIC and MBIC values of PSSAg were obtained at least three times independently; no differences were observed among the experiments.

bacterial strain	MIC [µg/mL]	MBIC [µg/mL]

*E. coli ATCC 23546*	0.76	1.14
*P. aeruginosa ATCC 10145*	0.19	0.76
*S. aureus* ATCC 29213	1.14	3.04
*S. epidermidis* ATCC 12228	0.76	0.76

The determined minimum inhibitory concentration (MIC) values confirmed that the studied PSSAg beads exhibit considerable antimicrobial activity. The MIC values for all of the tested bacterial strains are in the range from 0.76 to 1.14 µg/mL. The least susceptible bacterial strain was gram-positive *S. aureus*, for which the growth was inhibited at the concentration of 1.14 µg/mL. This result is consistent with previous reports on non-incorporated silver nanoparticles, which exhibit pronounced antibacterial activity towards gram-negative species. It has been suggested that the gram-negative species may be more susceptible to Ag penetration, as silver nanoparticles are able to more effectively interact with the cell components [42–43]. On the other hand, in the gram-positive strains the cell wall is thicker and the peptidoglycan is more cross-linked, which hampers the access of Ag into the cell [44]. Also, in our study the gram-negative species, especially *P. aeruginosa*, were more susceptible to the examined samples than the gram-positive *S. aureus*.

Biofilm formation is a strategy of microorganisms to avoid unfavorable environmental conditions. Due to high resistance of these microbial populations to commonly used therapeutics, biofilms are a substantial source of antibiotic failure and persistent infections [45]. The efficacy of the nanobeads in the inhibition of biofilm formation was estimated. As can be seen in Table 1 the minimum biofilm inhibitory concentration (MBIC) values are in the range from 0.76 to 3.04 µg/mL. These observations confirm a strong activity of PSSAg against biofilms compared to non-incorporated silver nanoparticles. Radzig and co-workers observed that Ag nanoparticles of 8.3 ± 1.9 nm in size hamper the biofilm formation of *E. coli* and *P. aeruginosa.* The reduction of bacterial biomass in the biofilm was visible when the concentration was higher than 5 μg/mL for *E. coli* and 10 μg/mL for *P. aeruginosa* [46]. Roe and co-workers examined the efficacy of nanosilver (average diameter of 10 nm) as an anti-biofilm agent used to coat the surface of catheters. The authors observed almost complete suppression of biofilm formation by *E. coli* and *S. aureus.* More than 50% inhibition was noted in the case of coagulase-negative staphylococci and *P. aeruginosa* [47].

In our work, the minimum concentrations of PSSAg beads inhibiting the biofilm formation are generally about two times higher than the MICs for the studied bacterial strains (with the exception of *S. epidermidis*). This is consistent with previous reports indicating a profound resistance of bacteria grown in biofilms to antimicrobials. Bacterial biofilms are generally less susceptible to silver nanoparticles than planktonic cells, probably due to the extracellular matrix coating the cells in a biofilm, aggregation of the cells to reduce their exposed surface and the retardation of the nanoparticle diffusion caused by aggregation [48]. Some species develop less complicated biofilm structures or even are not capable to form biofilms. The *S. epidermidis* strain used in our experiments probably does not form complex biofilm structures, as the three other bacterial strains [49]. In consequence the determined MIC and MBIC values for *S. epidermidis* are comparable.

**Figure 8:**
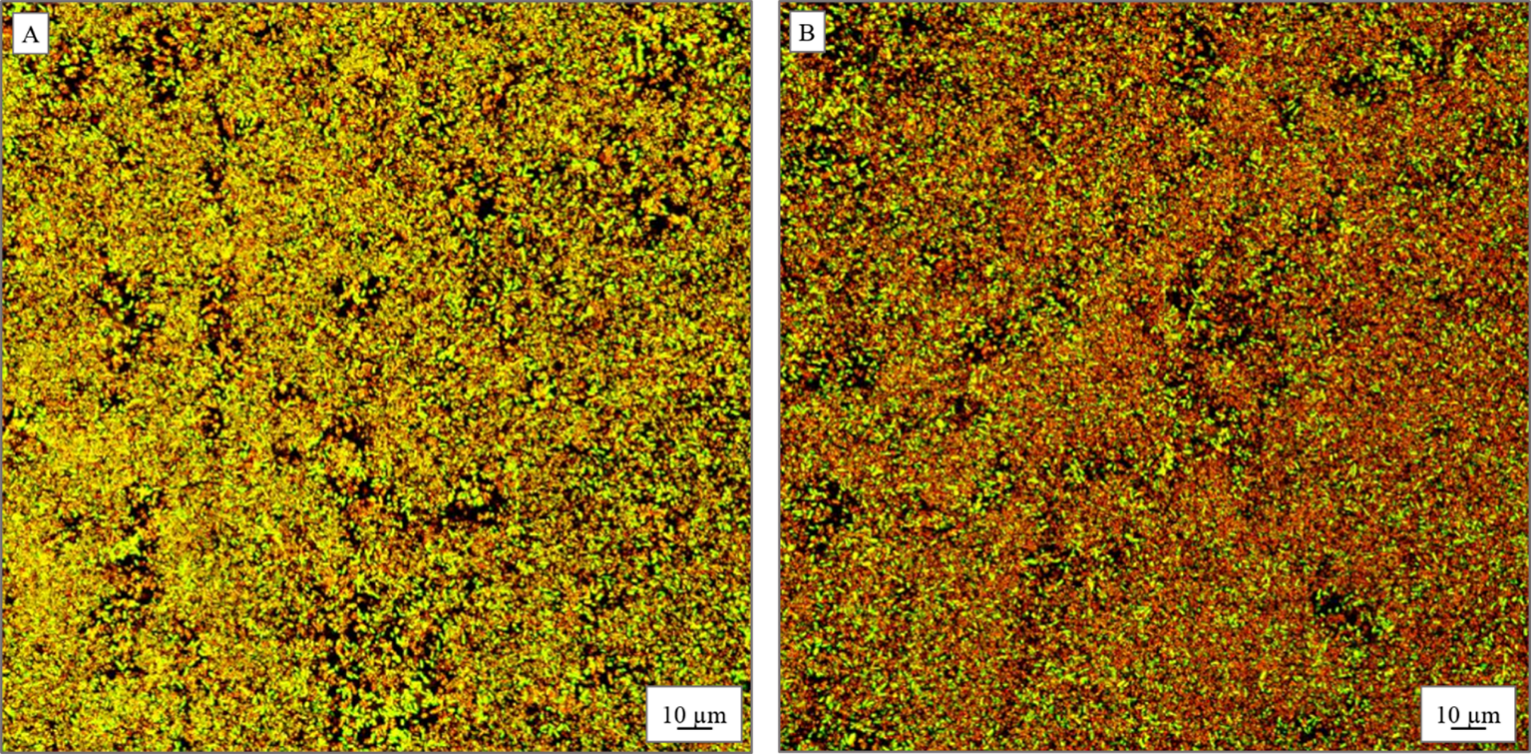
Confocal microscopy images of *P. aeruginosa* cells in biofilms (A) without PSSAg nanobeads and (B) incubated with 0.5 µg/mL PSSAg. Green cells are viable, red cells are dead.

**Figure 9:**
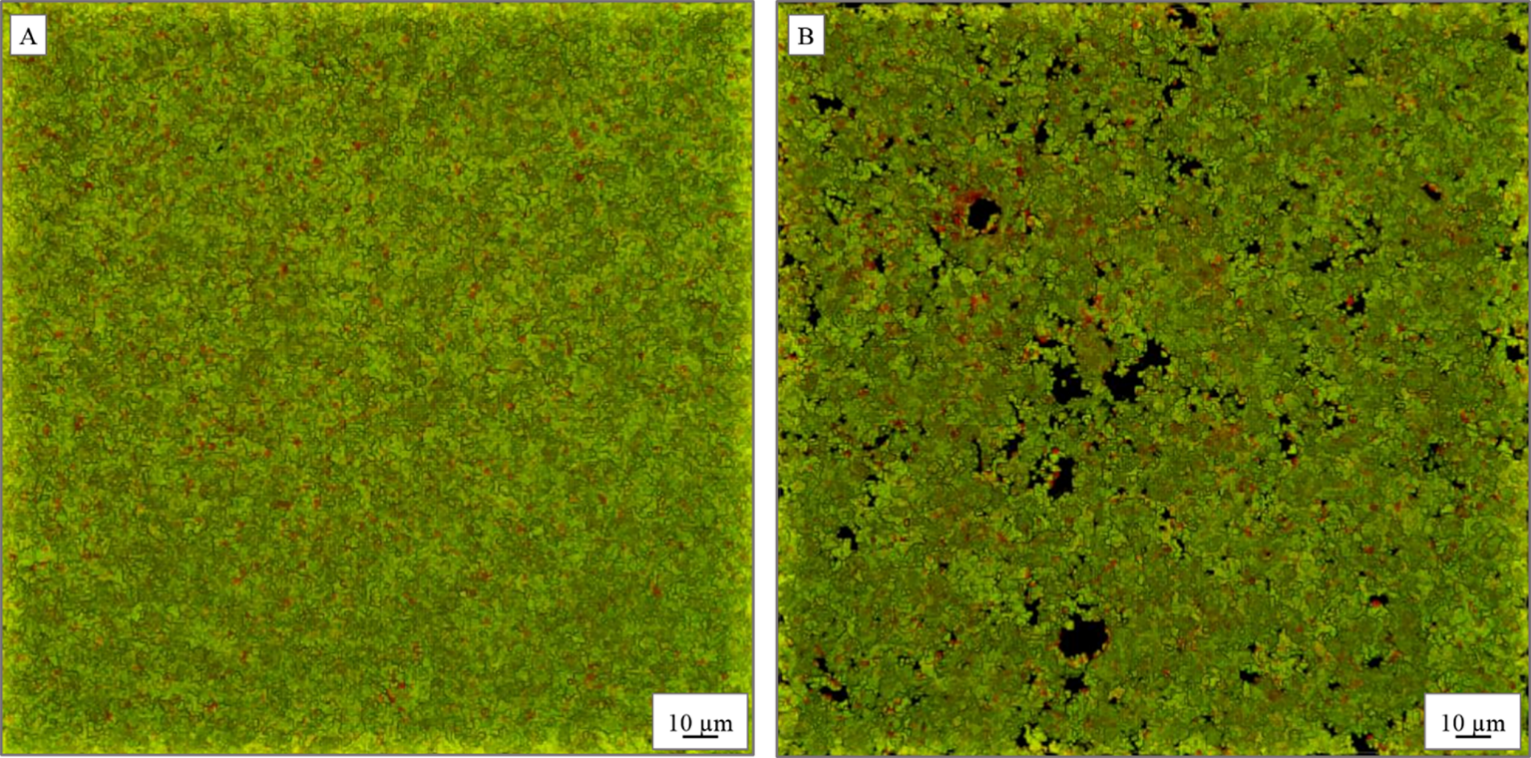
Confocal microscopy images of *S. aureus* cells in biofilms incubated (A) without nanocomposite, (B) with 0.5 µg/mL PSSAg. Green cells are viable, red cells are dead.

Further analysis of the bacterial viability in the presence of the nanobeads was investigated for the selected bacterial strains, gram-negative *P. aeruginosa* and gram-positive *S. aureus*. The analyses were conducted using the LIVE/DEAD BacLight bacterial viability kit and the samples were imaged with confocal fluorescence microscopy [50]. The test uses the properties of fluorescent dyes, namely, green SYTO 9 and red propidium iodide. The SYTO 9 stain labels the bacteria with intact membranes and those with damaged membranes. In contrast, propidium iodide penetrates only the bacteria with damaged membranes, thereby reducing the fluorescence of SYTO 9 when both dyes are present. The living cells appear green while the dead cells are red in the images of the biofilms. One can see that the treatment of *P. aeruginosa* resulted in a noticeable decrease of survival of bacterial cells. The incubation with 0.5 µg/mL of PSSAg nanobeads resulted in the death of 93% cells in the biofilm (Figure 8B), while the value for the control sample (Figure 8A) was 90%. On the other hand, for the *S. aureus* biofilm (Figure 9) the percentage of the red-stained cells is 10% (Figure 9B), whereas in the control sample this value is only 2% (Figure 9A). This low percentage is likely due to higher resistance of these species compared to *P. aeruginosa.* Regardless of this fact one can observe considerable antimicrobial activity of the nanobeads for both strains. One can also notice that for the *S. aureus* biofilm only small clusters of dead cells, close to the spaces and interruptions in the biofilm, structure are seen. These changes in the structure of the biofilm were not present in the control sample. Presumably some of the nanobeads sedimented on the bottom of the plate and prevented the formation of the nearby biofilm.

The above data clearly show that the silver-decorated nanobeads exhibit considerable antimicrobial properties, comparable or superior to those of non-incorporated silver nanoparticles. Polystyrene sulfonate is known to be non-toxic and it was shown to have no effect on the viability of various bacterial strains, including *S. epidermidis* [51]. Thus, it seems that the low values of MIC and MBIC are not associated directly with biocidal properties of the polymer itself. Instead, we may speculate, that the encapsulation of the silver nanoparticles in polymeric beads facilitates their access to bacterial cells. However, regardless of the detailed mechanism of the antimicrobial action, our data suggest the potential of the nanobeads as an active component in antiseptic materials.

## Conclusion

Gel-shell nanobeads modified through the incorporation of silver nanoparticles have been prepared in a multistep synthetic procedure. In the first step a hydrogel shell is fabricated around the polystyrene core in a sulfonation reaction. Then, silver ions are accumulated in the gel layer of the beads followed by reduction with sodium borohydride. The reduction reaction results in the generation of silver nanoparticles but also in a conversion of a fraction of sulfonic groups to thiol moieties. The thiol groups interact with the surface of silver nanoparticles anchoring them in the gel shell of the nanobeads, which likely stabilizes these structures. The hybrid particles reveal considerable antibacterial properties. This has been evaluated based on the determination of MIC and MBIC values and an estimation of bacterial viability through fluorescence staining.

## Experimental

### Chemicals

All chemicals were of the highest quality commercially available and were used as received: divinylbenzene (DVB)-cross-linked polystyrene latex beads (Magsphere), sulfuric acid (POCh, 95–97%), silver nitrate (Aldrich, 99%), sodium borohydride (Aldrich, ≥96%), polyvinylpyrrolidone (Aldrich, *M*_w_ ≈ 55000), sodium hydroxide (reagent grade, Chempur), buffer solutions (consisting of NaOH, H_3_PO_4_, H_3_BO_3_, CH_3_COOH) pH 3–10 (POCh), 0.1% crystal violet solution (Aldrich). Aqueous solutions were prepared using high-purity water (Milli-Q Plus).

For antibacterial tests, 96-well polystyrene microtiter plates (Greiner bio-one) and 40 mm glass bottom dishes (WillCo Wells; thickness of glass bottom: 0.16–0.19 mm) have been used. Microbial strains were chosen from the American Type Culture Collection (ATCC): *E. coli* ATCC 23546, *P. aeruginosa* ATCC 10145, *S. aureus* ATCC 29213, *S. epidermidis* ATCC 12228. The cultures were grown in Mueller–Hinton broth (MH; Biocorp Poland). The medium was supplemented with glucose when required (Chempur, Poland). For the analysis of bacterial viability the LIVE/DEAD BacLight bacterial viability kit (Promega) was used.

### Instrumentation

#### Microscopy

Scanning electron microscopy and electron microprobe (EDS) analysis were performed with a Zeiss Merlin field-emission SEM instrument. Transmission electron microscopy images were collected with a Zeiss Libra 120 EFTEM. Confocal laser scanning microscopy (CLSM) imaging of bacterial biofilms was performed with a Nikon AIR MP microscope equipped with a 60×, NA 1.4, oil immersion phase-contrast objective (excitation wavelength 488 nm).

#### Thermogravimetric analysis

Thermogravimetric analysis was performed with a TGA Q50 (TA Instruments). The measurements were performed under a nitrogen/oxygen atmosphere.

#### Combustion elemental analysis

The elemental analysis was conducted using a CHNS Analyzer VARIO EL III.

#### Zeta potential measurements

The measurements were carried out using a Zetasizer Nano ZS (Malvern). An aqueous suspension of the investigated particles was added to the buffer solution (pH 3–10). Each measurement was repeated three times. The zeta potential was calculated using the Smoluchowski equation.

#### Spectroscopy

Fourier-transform infrared data were collected with a Nicolet 6700 Continuum FTIR microscope (Thermo Electron Corporation). The chemical composition of the structure was examined using X-ray photoelectron spectroscopy (Kratos Analytical Axis Ultra DLD).

### Experimental procedures

**Preparation of sulfonated polystyrene beads and modification with silver nanoparticles:** 50 µL of a polystyrene bead suspension (10% w/w) was allowed to dry for 30 min at 70 °C. Then, 500 µL of concentrated H_2_SO_4_ was added to the dried sample and kept in a heated bath at 55 °C for 4 h. After this time, the sulfonated polystyrene spheres were separated by centrifugation (13400 rpm, 12100*g*, 5 min), and washed with deionized water several times, until a neutral pH value of the supernatant was achieved. The next step in the synthesis was the incorporation of silver ions within the sulfonated nanobeads. The beads were mixed with 150 µL aqueous AgNO_3_ solution (0.0125 M) for ca. 30 min followed by the addition of 150 µL of a freshly prepared aqueous solution of NaBH_4_ (0.075 M) and PVP (40 mg/mL). Subsequently, this mixture was kept at 55 °C and stirred using ultrasound for 30 min. The final product was collected by centrifugation (13400 rpm, 12100*g*, 5 min), and washed several times with deionized water.

**Preparation of silver nanoparticles (non-incorporated in polymer beads):** 150 µL of a freshly prepared solution of NaBH_4_ (0.075 M) in an aqueous solution of PVP (40 mg/mL) was added to an aqueous solution of AgNO_3_ (0.0125 M). Then, the mixture was kept at 55 °C and stirred using ultrasound for 30 min. The obtained product was collected by centrifugation (13400 rpm, 12100*g*, 5 min), and washed several times with deionized water.

**Antibacterial activity:** The antibacterial activity was studied by the determination of the minimum inhibitory concentration (MIC) of the samples using a broth microdilution technique performed in 96-well microtiter plates according to the standards of the Clinical and Laboratory Standards Institute (CLSI) [52]. Briefly, a series of twofold dilutions of the stock nanobeads solution (1.6 mg/mL) were prepared in MH broth to form a range of concentrations from 1 to 512 µg/mL (mass of the nanocomposite per volume). The samples were then inoculated with tested bacterial cultures providing a final cell density of 1 × 10^6^ CFU/mL (CFU: colony-forming units) and the plates were incubated statically at 37 °C for 24 h. The lowest nanobead concentration that resulted in no visible turbidity was considered as the MIC value. MBIC values were determined as described elsewhere [43]. The bacterial cultures were diluted 1:100 in fresh medium and then mixed with MH supplemented with 0.45% glucose and different concentrations of nanobeads in the wells of the microtiter plate (the final cell density was 1 × 10^6^ CFU/mL). After static incubation at 37 °C for 24 h, the lowest concentration of the nanobeads that inhibited biofilm growth, as determined by crystal violet staining [53], was taken as the MBIC. Each determination was performed in triplicate.

Confocal laser scanning microscopy was used to assess the bacterial viability in biofilms using the LIVE/DEAD BacLight bacterial viability kit. Overnight cultures of *P. aeruginosa* and *S. aureus* were 100-fold diluted in MH supplemented with 0.45% glucose and MBIC of 0.5 µg/mL of the nanobeads. Then the biofilms were grown on glass microscope dishes for 24 h at 37 °C. After incubation, the liquid medium was removed and the biofilms were gently rinsed with distilled water twice. Bacterial cells were stained with SYTO-9 and propidium iodide fluorophores according to the protocol and incubated in the dark for 30 min before the CLSM analyses. The reconstructions of the biofilm images were performed using the NIS-Elements interactive software. The percentage of red-stained cells was quantified with the *BioFilmAnalyzer* software.
